# Macrolide antibiotics exert antileukemic effects by modulating the autophagic flux through inhibition of hERG1 potassium channels

**DOI:** 10.1038/bcj.2016.32

**Published:** 2016-05-13

**Authors:** S Pillozzi, M Masselli, L Gasparoli, M D'Amico, L Polletta, M Veltroni, C Favre, G Basso, A Becchetti, A Arcangeli

**Affiliations:** 1 Department of Experimental and Clinical Medicine, University of Florence, Florence, Italy; 2 DI.V.A.L. Toscana s.r.l., Sesto Fiorentino, Italy; 3 Department of Pediatric Hematology Oncology, Azienda Ospedaliero-Universitaria Meyer, Florence, Italy; 4 Department of Women's and Children's Health, Clinic of Pediatric Hemato-Oncology, University of Padua, Padua, Italy; 5 Department of Biotechnology and Biosciences, University of Milano-Bicocca, Milan, Italy

Macrolide antibiotics (MAs) have a wide spectrum of activities against Gram-positive bacteria, but they have also been proposed as anticancer drugs for multiple tumor types.^1^ On these bases, clinical trials have been started, and their results evidenced clinical benefits.^1^ MAs have also been tested in hematologic malignancies, alone or in combination with chemotherapeutic drugs or tyrosine kinase inhibitors.^2, 3^ How MAs exert antineoplastic activity is unclear. Different mechanisms have been proposed, including modulation of autophagy.^4, 5, 6^

We tested the activity of two MAs, clarithromycin (Cla) and erythromycin (Er), on acute leukemia (AL) cells, both myeloid and lymphoid. Both MAs induced leukemia cell death in our cell lines. Cla was efficacious in AML cells, whereas the LD_50_ (dose lethal to 50% of animals tested) values of Er, although higher compared with Cla, were generally lower in ALL cells (representative IC_50_ (half-maximal inhibitory concentration) values of both MAs are shown in Figure 1a, whereas all the data are reported in Supplementary Table S1).

Next, we tested whether MAs affected the autophagic process of AL cell. Cla-induced vacuoli formation (Supplementary Figure S1) and the conversion of the light chain enhancer 3 (LC3) from its soluble (LC3I) to the membrane-bound form LC3II, in FLG 29.1 AML cells (Figure 1b left panel and Supplementary Figure S2). Both the effects indicate triggering of autophagy. Similar results were obtained after treatment of 697 ALL cells with Er (Figure 1b right panel and Supplementary Figure S2). The autophagic flux was determined by flow-cytometry, measuring cyto-ID-stained autophagic compartments through the Cyto-ID Green Detection Reagent (Supplementary Information). Cla induced an increase in Cyto-ID fluorescence in FLG 29.1 cells (Figure 1c, upper panel). This indicates an impairment in autophagic flux. Similar effects were produced by Er on 697 cells (Figure 1c, lower panel).

Autophagy is regulated by different signaling pathways: the PI3K/Akt pathway, which inhibits autophagy by interacting with mTORC1, and the Erk1/2 pathway, which activates autophagy and promotes cell survival.^4, 7^ Cla determined an early decrease in both Akt and Erk1/2 phosphorylation (Figure 1d and Supplementary Figure S2). The former effect is consistent with autophagy activation, whereas the latter points to inhibition of prosurvival signals and hence induction of apoptosis. Consistently, Cla induced the activation of caspase 3 in FLG 29.1 cells (Figure 1e and Supplementary Figure S2). Overall, MAs stimulate autophagy in AL cells through the inhibition of Akt, but they subsequently block the autophagic flux and induce autophagic cell death. In parallel, MAs also inhibit the Erk-dependent survival signals, thus triggering apoptosis. In other words, MAs activate both autophagic and apoptotic cell death in AL cells.

MAs can block hERG1 currents in reconstitued cellular models.^8, 9^ Conversely, blocking hERG1 has antileukemic effects both *in vitro* and *in vivo*.^10, 11, 12^ Therefore, we hypothesized that the effects of MAs on AL cells could depend on hERG1 inhibition. We first evaluated the effects of MAs on hERG1 currents of AL cells, using Cla and the AML cell line FLG 29.1 as a model (details are given in Supplementary Figure S3). Cla inhibited hERG1 currents in a concentration-dependent manner (IC_50_=38.5±7.0 μm). It also reduced hERG1B currents (IC_50_=66.8±9.8 μm), that is, the main hERG1 isoform expressed in leukemia cells.^13, 14^ Therefore, Cla blocks hERG1B with high efficacy, differently from classical hERG1 blockers like E4031.

Next, we studied the effects of hERG1 blocking (treatment with E4031) and silencing on the autophagic process. For the latter purposes, the level of autophagy in hERG1-silenced FLG 29.1 cells (FLG 29.1-sh7, whose characterization is shown in Supplementary Figure S4) was compared with that of cells infected with the empty vector (FLG 29.1 pLKO). hERG1 blockade induced a time-dependent increase in the percentage of cells with vacuoles (Supplementary Figure S1), paralleled by increased levels of LC3II (Figure 1f left panel and Supplementary Figure S2). Similar effects were obtained with the hERG1B-selective blocker, CD160130^(ref. 12)^ (Figure 1f right panel and Supplementary Figure S2). Moreover FLG 29.1 cells treated with E4031 showed an increase of AAF (Figure 1g). Hence, blocking hERG1 triggers autophagy and blocks the autophagic flux, similarly to MAs. Consistently, FLG 29.1-sh7 cells showed an increased percentage of cells with vacuoli (Supplementary Figure S1), increased levels of LC3II (Figure 1h and Supplementary Figure S2), but unchanged levels of MFI relative to the cyto-ID-stained autophagic compartments, compared with FLG pLKO cells (mean 258±7 vs 266±11; Figure 1i). Hence, silencing hERG1 triggers autophagy but, at difference with hERG1 blockade, does not impair the autophagy flux. Consistently, blocking hERG1 with E4031 or silencing its expression both induced a decrease in Akt and Erk 1/2 phosphorylation (Figure 1j and Supplementary Figure S2), but only E4031 led to activation of caspase 3, which triggers apoptotic cell death (Figure 1k and Supplementary Figure S2). Overall, these data confirm the hypothesis that hERG1 modulates the autophagic flux in AL cells, and that pharmacologically blocking the channel triggers autophagy, but blocks the autophagic flux, thus inducing both autophagic and apoptotic cell death, in analogy to MAs.

Finally, we tested whether the effects of MAs on AL cell death was related to their blocking effect on hERG1 currents. MAs were tested on hematopoietic cells that do not express the hERG1 channel: normal peripheral blood mononuclear cells (PBMNCs), and EBV infected B lymphocytes (EBV-B).^11^ Cla poorly affected both PBMNCs and EBV-B cells, showing LD_50_ values higher than 200 μm (Supplementary Figure S5). Cla had significantly (*P*<0.01) higher LD_50_ values in FLG 29.1-sh7 than in FLG 29.1-plkO (Supplementary Figure S5). This is clear from Figure 1l, showing the data obtained with 52 μm Cla in silenced and control cells. Finally, we tested the combination of MAs and E4031 on AL cell death. If added in conjunction with E4031, then both Cla and Er showed a larger effect on cell death (Figure 1m). This is consistent with the fact that MAs block hERG1 currents by binding a site different from the one targeted by E4031.^8^ Overall, we conclude that the antileukemic effect of MAs is totally or partially mediated by their blockade of hERG1 currents.

We next tested the effects of MAs (both Cla and Er) in combination with chemotherapeutic drugs commonly used to treat either ALL (Doxorubicin, Doxo) or AML (Cytarabine, Cyt). AL cells were cultured either in suspension or onto MSC cells, which are known to protect leukemia cells from chemotherapy-induced cell death (Pillozzi *et al.*^11^ references therein). Figures 2a and b show that the addition of LD_50_ concentrations of either MA in combination with chemotherapeutic drugs increased the percentage of Annexin V+/propidium iodide (PI)− cells. The effect was particularly evident in MSC-supported cultures. These results are corroborated by the CI values (Supplementary Table S2): MAs are synergic with chemotherapeutic drugs, especially when added to MSC-supported cultures. The combination of Er with prednisone (Pdn, 5 μm^11^ in 697 ALL cells significantly increased the percentage of Annexin V+/PI− cells, especially in MSC-supported cultures (Figure 2c). Finally, Cla in combination with Cyt (at 45 nm), also produced a significant increase in the percentage of Annexin V+/PI− cells in three primary AML samples co-coltured with MSC (Figure 2d).

The antileukemic effect of MAs, alone or in combination with chemotherapeutic drugs, was then evaluated *in vivo*, in both AML (HL60-*luc2*) and ALL (REH) mouse models. Details of both models are given in Supplementary Information. Cla decreased the AML burden compared to both untreated and Cyt-treated mice (Figure 1e, see also the median values of c.p.m. for each treatment group at different time points, in the right panel). Er reduced both bone marrow (BM) engraftment and PB invasion of ALL cells (Figure 1f), similarly to what was obtained with E4031 (Supplementary Figure S6).

Either MAs were then tested in combination with chemotherapeutic drugs: mice injected with HL60 AML cells were treated with Cla and Cyt, whereas mice injected with REH ALL cells (notoriously resistant to corticosteroids^11^) were treated with Er and the corticosteroid dexamethasone (Dexa). Combination treatment of Cla with Cyt significantly increased the overall survival of HL60-*luc2* injected mice compared with control group (*P*=0.0024; Figure 1g). Combination treatment of Er with Dexa improved overall survival, (Figure 1h), reduced BM engraftment and roughly abolished PB leukemia burden compared with Dexa treatment (inset to Figure 1h).

In this paper, we provide evidence that (1) MAs have antileukemic activity, either *in vitro* or *in vivo*, in both AML and ALL, alone or in combination with chemotherapeutic drugs, (2) these effects depend on a complex modulation of both autophagy and intracellular signaling pathways regulating cell survival and apoptosis, and (3) are mediated by hERG1 channels. Compared with hERG1 blockers, MAs have a low risk of inducing torsade-de-points cardiac arrhythmias.^9^ We thus propose to include these compounds in treatment schedules of resistant acute leukemias in combination with chemotherapeutic drugs.^15^

## Figures and Tables

**Figure 1:**
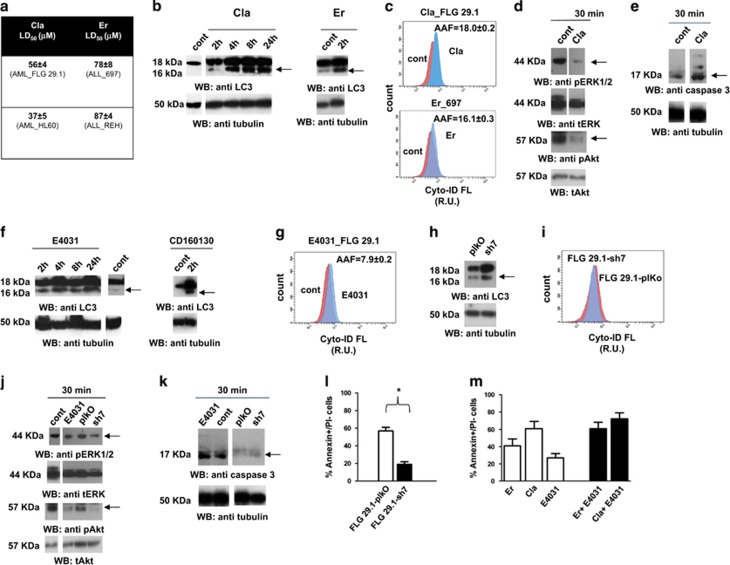
Effects of MAs Clarithromycin (Cla), Erythromycin (Er) and hERG1 blockade and silencing on autophagy in acute leukemia cells. (**a**) LD_50_ values of Cla and Er in a panel of myeloid and lymphoid (AML cells: FLG 29.1, HL60; B cell precursor (BCP)-ALL cells: 697, REH) cell lines. Cells were treated with different concentrations of Cla and Er for 48 h and analyzed through the Annexin V/PI test. LD_50_ values were evaluated by nonlinear regression analysis using Origin 6 software (Microcal Software). Values are mean±s.e.m. of three indipendent experiments each performed in triplicate. (**b**) Left panel. Western blot of light chain enhancer 3 (LC3) expression (18 and 16 kDa bands) in FLG 29.1 cells treated with 56 μm Cla (the LD_50_ value as shown in **a** and in Supplementary Table 1S) or for different time points. LC3 was determined, as a biomarker of the autophagic process. Reprobing of the membrane was with an anti-tubulin antibody. Densitometric analysis is reported in Supplementary Figure S2. Right panel. Western blot of LC3 expression (18 and 16 kDa bands) in 697 cells treated with 78 μm Er (the LD_50_ value as shown in Figure 2) for 2 h. Membrane reprobing as above. Densitometric analysis is reported in Supplementary Figure S2. (**c**) Cyto-ID flow-cytometry analysis (representative panels) of FLG 29.1 and 697 cells treated with Cla (upper panel) and Er (lower panel) at their LD_50_ value for 2 h. The method is detailed in Materials and Methods. The mean autophagy activity factor (AAF) value, calculated in three separate experiments from AAF= 100 × [(MFI-treated cells−MFI-untreated cells)/MFI-treated cells] is shown on the top of each panel. (**d**) Western blot of pERK1/2 (top) and pAkt (bottom) in FLG 29.1 cells treated for 30 min with 56 μm Cla. Membrane reprobing (with anti-ERK1/2 and total Akt antibodies) as in **b**. Densitometric analysis is reported in Supplementary Figure S2. (**e**) Western blot of caspase 3 in FLG 29.1 treated for 30 min with 56 μm Cla. Membrane reprobing as in **b**. Densitometric analysis is reported in Supplementary Figure S2. (**f**) Left panel. Western blot of LC3 expression in FLG 29.1 cells treated with 22 μm E4031 for different time points: hERG1 blockade by E4031 induced increased levels of LC3II at least 2 h after treatment and last at least up to 24 h. Membrane reprobing was performed as in **b**. Densitometric analysis is reported in Supplementary Figure S2. Right panel. Western blot of LC3 expression in FLG 29.1 cells treated with CD160130, at the LD_50_ value (3.5 μm) for 2 h. Membrane reprobing and was performed as in **b**. (**g**) Cyto-ID flow-cytometry analysis (representative panels) of FLG 29.1 in control conditions and after 2 h treatment with 22 μm E4031. The AAF value, calculated as in **c** is reported on the top. Data shown are representative of two independent experiments. (**h**) Western blot of LC3 expression in FLG 29.1-sh7 and FLG 29.1-plKo. Membrane reprobing was performed as in **b**. Densitometric analysis is reported in Supplementary Figure S2. (**i**) Cyto-ID flow-cytometry analysis of FLG 29.1-sh7 and FLG 29.1-plKo cells. A representative panel is shown. MFI values from two independent experiments are reported in the text. (**j**) Western blots of pERK1/2 (top) and pAkt (bottom) of FLG 29.1-sh7, FLG 29.1-plKo and FLG 29.1 cells treated or not with 22 μm E4031 for 30 min. Membrane reprobing was performed as in **b**. Densitometric analysis is reported in Supplementary Figure S2. (**k**) Western blot of caspase 3 of FLG 29.1-sh7, FLG 29.1-plKo and FLG 29.1 cells treated or not with 22 μm E4031 for 30 min. Membrane reprobing was performed as in **b**. Densitometric analysis is reported in Supplementary Figure S2. (**l**) Percentage of Annexin V+/PI− cells in FLG 29.1-plkO and FLG 29.1-sh7 cells treated with 52 μm Cla for 48 h (*P*<0.01, Student's *t-*test). (**m**) Percentage of Annexin V+/PI− cells in FLG 29.1-plkO cells treated with 52 μm Cla, or 78 μm Er, alone or in combination with 22 μM E4031. Values are mean±s.e.m. of two indipendent experiments each performed in triplicate.

**Figure 2:**
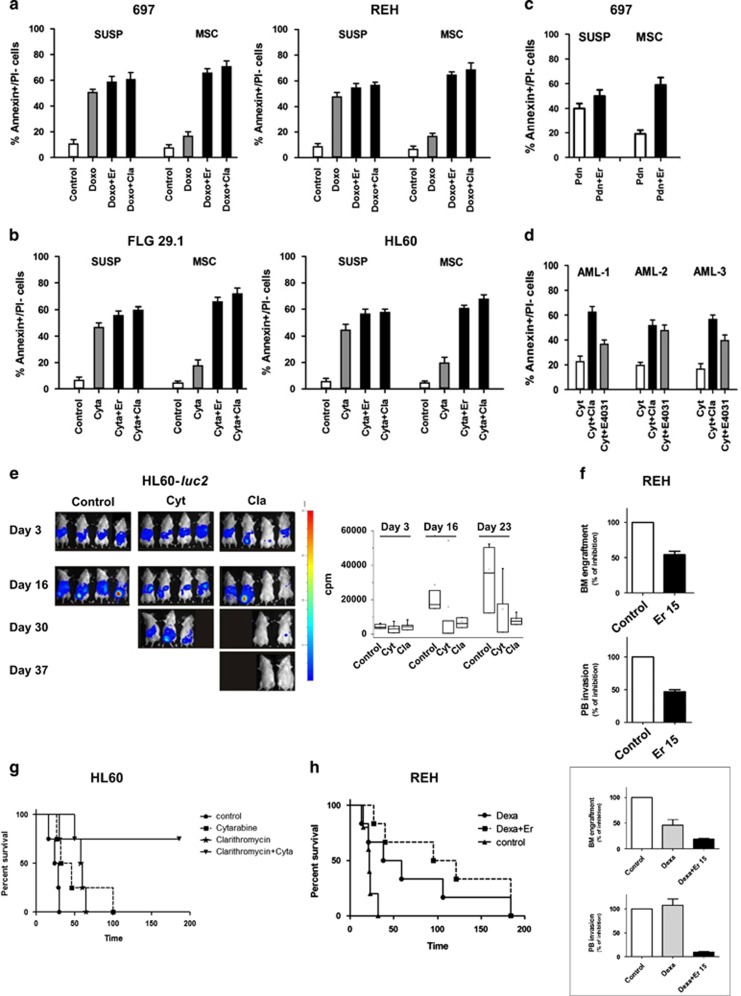
Effects of combination treatment with MAs and conventional antileukemic agents in ALL and AML cell lines *in vitro* and *in vivo*. A panel of leukemic cell lines were cultured with or without MSC (suspension) and exposed to LD_50_ of doxorubicin (Doxo; 0.1 μg/ml) or cytarabine (Cyt; 45 nm) with or without the corresponding LD_50_ dose of the MAs antibiotic Cla/Er for 48 h. (**a**) ALL cell lines (BCP-ALL: 697, REH) exposed to LD_50_ of Doxo in the presence of LD_50_ of Cla. (**b**) AML cell lines (FLG 29.1; HL60) exposed to LD_50_ of Cyt in the presence of LD_50_ of Cla. The percentage of Annexin V+/propidium iodide (PI)− cells was measured. Values are mean±s.e.m. of three indipendent experiments each performed in triplicate. (**c**) 697 cells were cultured with or without MSC and exposed to LD_50_ of prednisone (5 μm) with or without the LD_50_ dose of Er for 48 h. The percentage of Annexin V+/PI− cells was measured. Values are mean±s.e.m. of two indipendent experiments each performed in triplicate. (**d**) Three representative pediatric AML primary samples were cultured onto MSCs and treated with LD_50_ doses relative to FLG 29.1 of either Cyt (45 nm, see Supplementary Information), MA antibiotic Cla (56 μm) and E4031 (50 μm) for 48 h. The percentage of Annexin V+/PI− cells was measured. Values are mean±s.e.m. of one experiment performed in triplicate. Statistical analysis was carried out with the Student's *t-*test (AML-1: Cyt+Cla vs Cyt, *P*<0.01; AML-2: Cyt+Cla vs Cyt, *P*<0.01; AML-3: Cyt+Cla vs Cyt, *P*<0.01). (**e**) SCID mice were injected with HL60-*luc2* cell line (5 × 10^6^ cells intraperitonially (i.p.)) and starting from day 5, animals were treated daily for 14 consecutive days with saline (control, *n*=4), Cyt (6.25 mg/kg, i.p., *n*=4); Cla (15 mg/kg, by oral gavage, *n*=4). Images were acquired with Photo Acquisition software (Biospace Laboratory, Paris, France) and processed with M3 Vision software (Biospace Laboratory). Median values of counts per minutes (c.p.m.) reported for each group of treatment at different time points are shown in the right panel. (**f**) NOD SCID mice were inoculated with REH cells on day 0 and after one week treated for 2 weeks with saline (Con, *n*=4) and Er (Er15, 15 mg/kg, *n*=4) and sacrificed 3 weeks after cell injection. Leukemia BM engraftment and PB burden were evaluated by FACS analysis estimating the hCD45+/mCD45+ ratio and were reported as percentage of the control for each treatment group. (**g**) SCID mice were injected with HL60-*luc2* cell line (5 × 10^6^ cells i.p.) and starting from day 5, animals were treated daily for 14 consecutive days with saline (control, *n*=4), Cyt (6.25 mg/kg, ip, *n*=4); Cla (15 mg/kg, by oral gavage, *n*=4) and Cyt (6.25 mg/kg, i.p.) plus Cla (15 mg/kg, by oral gavage, *n*=4). Survival curves of each experimental group, estimated by Kaplan and Meier analysis are reported (*P*=0.0024). (**h**) NOD SCID mice were inoculated with REH cells on day 0 and treated for 14 consecutive days with saline (Con, *n*=5), Dexa (15 mg/kg, *n*=5) and Dexa (15 mg/kg) plus Er (15 mg/kg, *n*=5). Survival curves of Control, Dexa and Dexa+Er experimental group, estimated by Kaplan and Meier analysis are reported (*P*=0.208): median survival is 22 days in control group, 48.5 days in Dexa group and 108 days in Dexa+Er group. Inset. An additional group of mice (*n*=3 per each group of treatment, treated as reported above) were analyzed 3 weeks after cell injection and BM and PB collected. Leukemia BM engraftment and PB burden were evaluated by FACS analysis estimating the hCD45+/mCD45+ ratio and were reported as percentage of the control for each treatment group.
