# Supplementary material for: Macrolide antibiotics exert antileukemic effects by modulating the autophagic flux through inhibition of hERG1 potassium channels
Source: Blood Cancer J. 2016 May 13;6(5):e423–. doi: 10.1038/bcj.2016.32 (PMC4916301; doi:10.1038/bcj.2016.32)
Supplement: Supplementary Information [file bcj201632x1.docx]

**Supplementary Information**

**LETTER TO THE EDITOR**

**Macrolide antibiotics have an antileukemic effect by modulating the autophagic flux through the involvement of hERG1 potassium channels.**

**Supplementary Materials.**

**Cell cultures.** B cell precursor (BCP)-ALL cell lines (REH and 697), the T-ALL cell line Jurkat, AML cell lines (FLG 29.1 (AML-M5), HL60 (AML-M3) and K562 (AML)) and the EBV-infected B-lymphocytes were cultured in RPMI 1640 medium supplemented with 2mM L-glutamine, 10% bovine calf serum (HyClone). Human bone marrow–derived mesenchymal stromal cells (MSCs), immortalized by transduction with the human telomerase reverse transcriptase (hTERT), were maintained in RPMI 1640 medium supplemented with 2mM/L glutamine, 10% fetal bovine serum (HyClone), 10^-6^M hydrocortisone (Sigma-Aldrich), 100 U/mL penicillin, and 100 μg/mL streptomycin as described in (1). MSCs were seeded in 96-well flat-bottomed plates (Costar, Corning) coated with fibronectin (1 μg/well; Sigma-Aldrich) and grown until confluence. To prepare co-cultures of MSC and leukemic cell lines (or primary AML samples), MSCs were washed with AIM-V medium (Invitrogen) and leukemic cells, resuspended in AIM-V medium, were placed on the MSC layer at 1x10^5^ cells/well. MAs were used at the final concentrations indicated in Figure legends. Cyt was used at final concentration of 45 nM (2), Doxo of 0.1μg/ml (1) and Pdn 5 μM (1). Cultures were maintained for 48 hours at 37°C, 5% CO2, and 90% humidity. After incubation, cells were separated from MSCs by pipetting with ice-cold PBS and processed for apoptosis analysis as described in “Apoptosis analysis.”

**Cells transfection.** HL60 cells were transfected with firefly luciferase gene using pGL4.51[luc2/CMV/Neo] plasmid (Promega, Madison, WI) as described in (3). 3.5x10^6^ cells were mixed with 20 ng of plasmid and electroporated by Gene Pulser X cell Electroporation system (BioRad, US) following the manufacturer protocol. Stable cell lines were selected using G418.

For hERG1 stable silencing in FLG 29.1 cells, a commercially available TRC shRNA-library (Open Biosystems) was used. The shRNA construct included a hairpin of 21 base pair sense/antisense stem and a 6 base pair loop cloned into the pLKO.1 vector. Lentivirus viral particles were produced by co-transfecting the second generation lentiviral plasmids and the transfer vector construct into the HEK293T packaging cell line. Stable cell lines were selected using puromycin (1.5 μg/ml).

**Evaluation of autophagic vacuoles.** FLG 29.1, FLG 29.1-plKo and FLG 29.1-sh7 cells, untreated or treated with either MAs or E4031, were cytospun onto glass slides, fixed and stained with May Grunwald& Giemsa to evaluate the presence of vacuoles. The method and representative cytospin images from four independent experiments are shown in Supplementary Figure S1.

**Peripheral Blood Mononuclear Cell (PBMNC) Isolation**. PBMNCs were isolated from buffy coat preparation of two independent healthy donors, using the Ficoll-Hypaque gradient centrifugation. Mononuclear cells were recovered at the interphase, washed, counted, and then used in the apoptosis experiments.

**Patients.** Bone marrow (BM) samples from children with newly diagnosed AML were analyzed at the Hematology-Oncology Laboratory of the Department of Pediatrics, University of Padova. Diagnosis was made according to standard cytomorphology, cytochemistry, and immunophenotypic criteria. Patients studied were enrolled in the AIEOP-BFM ALL 2009 therapy protocol, approved by the local Ethical Committee (Comitato Etico per la Sperimentazione dell'Azienda Ospedaliera

di Padova, N. 0002862- 18/01/2012). Patient's parents or their legal guardians provided written informed consent following the tenets of the Declaration of Helsinki.

**Protein extraction and Western blotting (WB).** Protein extraction and WBs were performed as in (4). The following primary antibodies were used: anti-hERG1 rabbit polyclonal (DIVAL Toscana Srl, dilution 1:1000), anti-hERG1B rabbit polyclonal (Dival srl, dilution 1:1000), anti-phospho-p44/42 MAP kinase (Thr202/Tyr204) (Cell Signaling, dilution 1:500), the anti-pAkt1/2/3 (Thr308)R (Santa Cruz Biotechnologies, dilution 1:500), anti-caspase 3 (E-8) (Santa Cruz Biotechnologies, dilution 1:1000) and LC3 A/B (Abcam, diluition 1:1000). Anti-α-tubulin mouse monoclonal (Sigma, dilution 1:500), anti-tAkt1/2/3 (H-136) (Santa Cruz Biothecnologies, dilution 1:500), anti ERK1 (C-16) (Santa Cruz Biothecnologies, dilution 1:500) antibodies were used as loading controls. Western blotting images were acquired with an Epson 3200 scanner, and the relative bands were analyzed by Scion Image software (Scion Corporation).

**Apoptosis Analysis.** Apoptosis was analyzed through the Annexin V/propidium iodide assay (Annexin-VFLUOS staining kit; Roche Diagnostics, Mennheim, Germany). LD_50_ values were calculated by fitting the data points with a sigmoidal curve using Origin 6 software. The CalcuSyn software (version 2, Biosoft), based on the method of the combination index (CI) of Chou and Talalay (5), was applied to determine synergy/additivity/antagonisms relative to drug combinations. Synergy, additivity, and antagonism were defined by a CI < 1, CI = 1, or CI > 1, respectively.

**Cyto-ID Autophagy detection.** The Cyto-ID assay (Enzo Life Sciences) was used. It is based on Cyto-ID staining of autophagic compartments in live cells through the use of a specific dye that selectively stains autophagic compartments and therefore allows determination of autophagic flux as accumulation of stained compartments (6, 7). An increase in the number of autophagic vesicles, which stain green is detected as an increase in fluorescence in the FL-1 channel. Cells (FLG 29.1, FLG 29.1-plKo and FLG 29.1-sh7 cells) were seeded in triplicate (2.0 x 10^4^ cells/ml) and treated with Cla (56 μM) or E4031 (40 μM) for 2 hours. Cells were harvested and incubated in Cyto-ID (1 μl Cyto-ID/1ml cell culture medium without phenol red indicator) for 30 minutes and washed prior to analysis by flow cytometry FACScan. The mean autophagy activity factor (AAF) value for each experimental treatment was then determined as following: AAF= 100x [(MFI treated cells – MFI untreated cells)/MFI treated cells]. AAF is a measure as the difference (in terms of MFI) between the amount of the Cyto-ID dye accumulated within cells in the presence of an autophagy inducer and thus indicates the amount of autophagic flux.

**Patch clamp analysis.**

Recording of hERG currents. Membrane currents were recorded in the whole-cell configuration of the patch-clamp technique, at room temperature (about 25°C). Electrodes were pulled from borosilicate glass capillaries (inside diameter 0.86 mm, outside diameter 1.5 mm; Harvard Apparatus, Holliston, MA, USA), using a PC-10 pipette puller (Narishige, Tokio, Japan). Electrodes typically had a resistance of 3.5-5 MΩ. Series resistance was always compensated up to approximately 80%. Currents were amplified and filtered using a Axopatch-1D (Molecular Devices, Sunnyvale, CA) interfacing with a Digidata 1440A (Molecular Devices) and a computer with pClamp 10.3 software (Molecular Devices). Currents were low-pass filtered at 2 kHz and digitized online at 10 kHz. Data were subsequently analysed off-line with pClamp and Origin 8.0 (Microcal Inc., Northampton, MA, USA) software. Solutions. The pipette solution contained (in mM): K^+^ aspartate 130, NaCl 10, MgCl_2_ 2, CaCl_2_ 2, HEPES 10, EGTA 10, titrated to pH 7.3 with KOH. During the experiments, cells were usually perfused with an extracellular solution containing (in mM): NaCl 95, KCl 40, CaCl_2_ 2, MgCl_2_ 2, HEPES 10, Glucose 5, adjusted to pH 7.4 with NaOH. In this case, the potassium equilibrium potential (E_K_) was -30 mV. High extracellular [K^+^] was applied to increase the amplitude of inward hERG1 currents measured at -120 mV, thus avoiding the necessity of applying excessively negative test potentials.

Protocols. The current-voltage relationships for hERG channels were determined from measurements of peak tail current amplitude at -120 mV (for 1.1–s), following 15-s conditioning potentials (applied in 10 mV increments) from 0 mV to -70 mV. The time between consecutive trials in the same stimulating protocol was 4 s at 0 mV. The same stimulation protocol was used to determine the concentration-response relations for different drugs.

The same stimulation protocol was used to determine the concentration-response relationships for Clarithromycin (Cla). Each compound was applied for at least 3 minutes (or until steady-state inhibition was attained), with each concentration being tested on at least five different cells.

**I*n vivo* experiments.** Mice experiments were performed at the Laboratory of Genetic Engineering for the Production of Animal Models (LIGeMA) at the Animal House of the University of Florence (Ce.S.A.L.). All experiments on live vertebrates have been carried out in accordance with the Principles of Laboratory Animal Care (directive 86/609/EEC).

The number of animals necessary for the experiments has been calculated with the “resource equation” method (Jaykaran Charan and N. D. Kantharia, How to calculate sample size in animal studies? J Pharmacol Pharmacother. 2013 Oct-Dec; 4(4): 303–306). According to this method a value “E” is measured, which is the degree of freedom of analysis of variance (ANOVA). Though, this method is based on ANOVA, it is applicable to all animal experiments. Any sample size, which keeps E between 10 and 20 should be considered as an adequate. E can be measured by following formula: E = Total number of animals − Total number of groups. Moreover we foresee an attrition of about 20% (mortality after surgery and/or failing to engraft) and hence the final number per group has been determined.

Cla was tested in a AML model (i.e. HL60-*luc2* cells injected i.p. into SCID mice ) and Er in a ALL model (i.e. REH cells injected i.v. into NOD SCID mice).

AML mouse model. The AML cell line HL60 expressing luciferase (HL60-*luc2*) was injected intraperitoneally (i.p.) in 5 weeks old female SCID mice (5x10^6^ cells/mouse) as described in (3). Briefly, to track the HL60-*luc2* cells, bioluminescent optical imaging was performed weekly, using Photon Imager system (Biospace Lab, Paris, France), including a cooled charge-coupled device (CCD) camera. Bioluminescent images were acquired for a total 3 minutes exposure, in ventral position, 5 minutes after i.p. injection of D-luciferin (150 mg/Kg, XenoLight RediJect D-Luciferin, Caliper Life Sciences). Optical images were analyzed with M3 Vision software (Biospace Lab, Paris, France). Counts per minutes (CPM) values were determined for each single animal using ROI (region of interest) tool. Imaging at day 3 was used to randomize the mice in different treatment groups with an average CPM signal as homogeneous as possible. Imaging monitoring was effectuated from day 5 post cell injection and fourteen consecutive days lasting: mice were daily treated with saline, Cyt (6,25 mg/Kg, ip) or Cla (15 mg/Kg, by oral gavage (og)).

In a second set of experiment mice were injected with HL60 cells as above, treated for 14 consecutive days starting from day 5 with saline, Cyt (6,25 mg/Kg, ip), Cla (15 mg/Kg, by og) or Cyt (6,25 mg/Kg, ip) plus Cla (15 mg/Kg, by oral gavage (og)) and housed in sterile room to evaluate the overall survival (OS).

ALL mouse model. The ALL cell line, REH was used to test the effect *in vivo* of Er in immunodeficient mice (NOD SCID). In a first set of experiments, 5x10^6^ REH cells were injected via the tail vein (i.v. injection) and a week later, mice were daily treated, for fourteen consecutive days, with Er (Er 15, 15 mg/Kg and Er 60, 60 mg/Kg), E4031 (20 mg/kg) or saline. Mice were sacrificed 3 weeks after cell injection and leukaemia BM engraftment and PB burden were evaluated by FACS analysis estimating the hCD45/mCD45 ratio as in (1). In a second set of experiments NOD SCID mice were injected as above and treated for two weeks with dexamethasone (Dexa) (15 mg/kg), Dexa (15 mg/kg) plus Er (15 mg/kg) and saline. A group of mice was sacrificed 3 weeks after cell injection and leukaemia BM engraftment and PB burden was evaluated as reported above, while a group of mice was maintained untouched in the sterile room, to evaluate OS.

**Statistical analysis**

Graphs and statistical analyses were prepared using Prism 4.00 (Graph Pad). All values are presented as mean ± standard error of the mean. Statistical significance was measured by t-test: ∗P < 0.05. For all graphs P values are indicated in the corresponding figure legend.

**Supplemental References**

1. Pillozzi S, Masselli M, De Lorenzo E, Accordi B, Cilia E, Crociani O *et al*. Chemotherapy resistance in acute lymphoblastic leukemia requires hERG1 channels and is overcome by hERG1 blockers. *Blood*. 2011;**117(3)**: 902-914.
2. Kaiser M, Kühnl A, Reins J, Fischer S, Ortiz-Tanchez J, Schlee C, *et al*. Antileukemic activity of the HSP70 inhibitor pifithrin-μ in acute leukemia. *Blood Cancer J*. 2011;**1(7)**: e28.
3. Gasparoli L, D'Amico M, Masselli M, Pillozzi S, Caves R, Khuwaileh R *et al*. New pyrimido-indole compound CD-160130 preferentially inhibits the KV11.1B isoform and produces antileukemic effects without cardiotoxicity. *Mol Pharmacol*. 2015; **87(2)**: 183-196.
4. Pillozzi S, Brizzi MF, Bernabei PA, Bartolozzi B, Caporale R, Basile V *et al*. VEGFR-1 (FLT-1), beta1 integrin, and hERG K+ channel for a macromolecular signaling complex in acute myeloid leukemia: role in cell migration and clinical outcome. *Blood*. 2007;**110(4)**: 1238-1250.
5. Chou TC, Talalay P. Quantitative analysis of dose-effect relationships: the combined effects of multiple drugs or enzyme inhibitors. *Adv Enzyme Regul*. 1984;**22**: 27-55.
6. Guo S, Liang Y, Murphy SF, Huang A, Shen H, Kelly DF, Sobrado P, *et al*. A rapid and high content assay that measures cyto-ID-stained autophagic compartments and estimates autophagy flux with potential clinical applications. *Autophagy*. 2015;**11(3)**: 560-572.
7. Galluzzi L, Pietrocola F, Bravo-San Pedro JM, Amaravadi RK, Baehrecke EH, Cecconi F,*et al*. Autophagy in malignant transformation and cancer progression. *EMBO J.* 2015;**34(7)**: 856-880.

**Supplementary Figures**


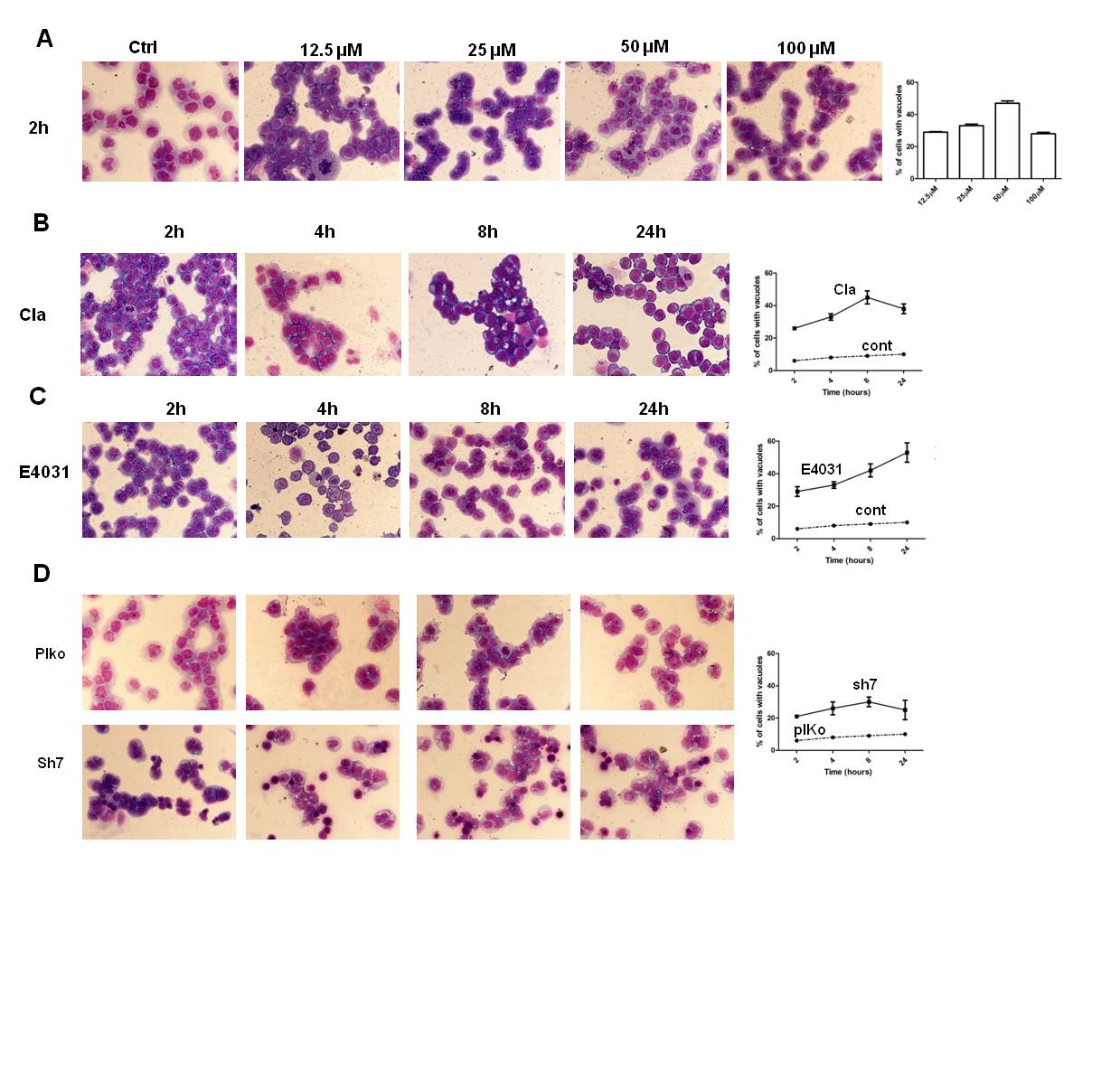


**Supplementary Figure S1. Effect of MA Cla, E4031 and hERG1 silencing on autophagy in acute leukemia cells.**

A) FLG 29.1 cells were treated with Cla at increasing concentrations (12.5-100 μM) for two hours and the percentage of cells with vacuoles was evaluated. Following the treatment, cells were cytospun onto glass slides and fixed and stained with May Grunwald Giemsa. Cells were observed at × 40 magnification using a light microscope. Vacuoles were counted in four different microscopic fields and the percentage of cells with vacuoles determined. Values in the graph are means±SEM of three indipendent experiments Cytospin images are representative of three independent experiments. Images were captured using a Nikon Eclipse E200 microscope equipped with the DS-SM-L1 digital sight camera system. B) FLG 29.1 cells were treated or not (control) with Cla (50 μM) for different time points (range 2-24 hours) and the percentage of cells with vacuoles was evaluated. Values in the graph are means±SEM of four indipendent experiments. C) Time course of the percentage of cells with vacuoles in FLG 29.1 cells treated or not (control) with 22 μM E4031. Values are means±SEM of three indipendent experiments. D) Time course of the percentage of cells with vacuoles in silenced leukemic cells FLG 29.1 (FLG 29.1-sh7) and FLG 29.1-plKo (control) cells. Values are means±SEM of three indipendent experiments.

**
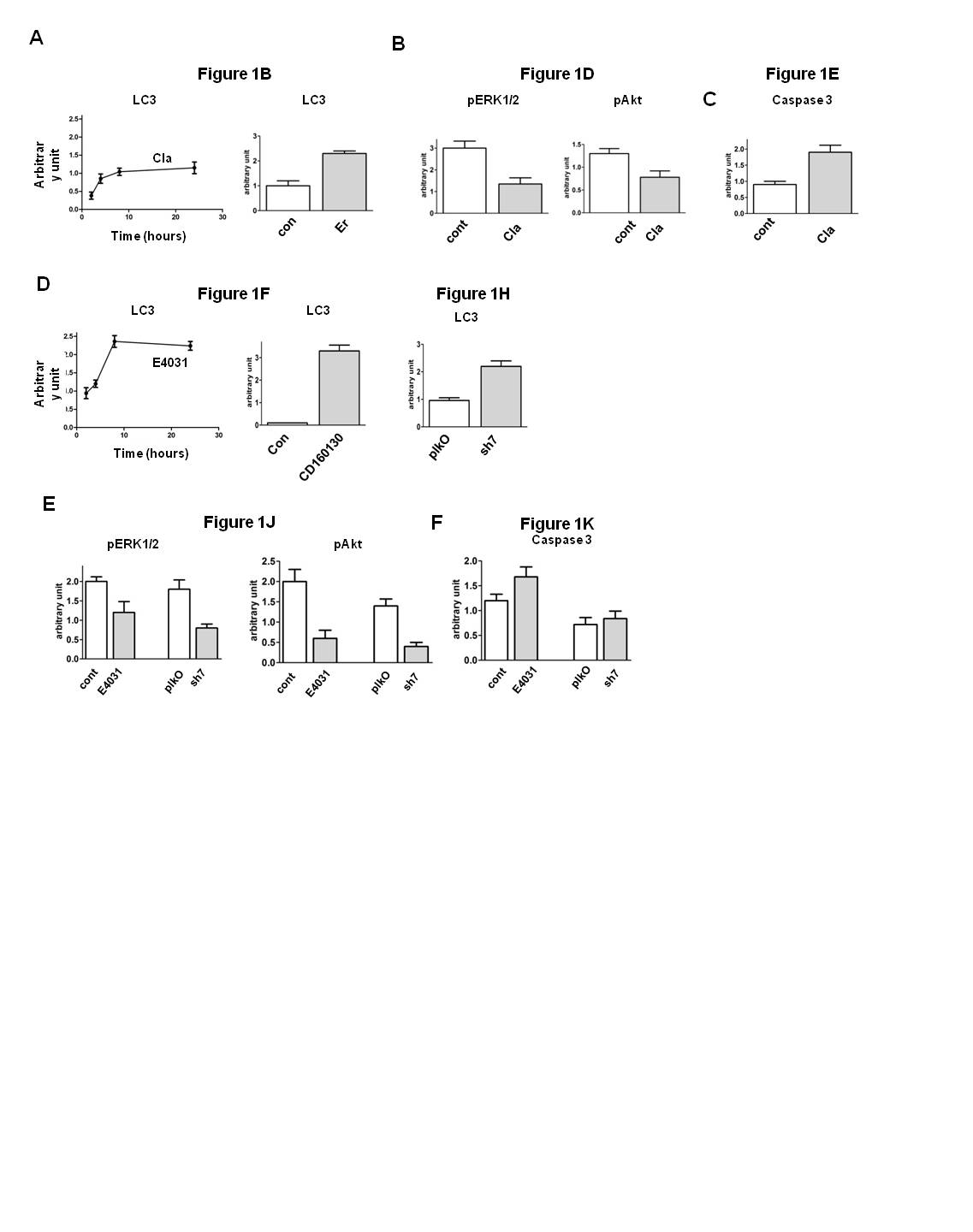
Supplementary Figure S2. Densitometric analysis relative to the activity of signalling proteins involved in the autophagic flux.** A) Densitometric analysis of LC3 proteins relative to experiments reported in Figure 1B. The intensity of the band relative to LC3 proteins was normalized to the intensity of the band of the tubulin protein. Analysis was performed with Scion Image software on three different experiments. B) Densitometric analysis of pERK and pAkt proteins relative to experiments reported in Figure 1D. The intensity of the band relative to pERK or pAkt proteins was normalized to the intensity of the band of the total ERK or total Akt protein. Analysis was performed with Scion Image software on two different experiments. C) Densitometric analysis of caspase 3 protein relative to experiments reported in Figure 1E. The intensity of the band relative to caspase 3 protein was normalized to the intensity of the band of the tubulin protein. Analysis was performed with Scion Image software on one experiment. D) Densitometric analysis of LC3 proteins relative to experiments reported in Figure 1F and H respectively. The intensity of the band relative to LC3 proteins was normalized to the intensity of the band of the tubulin protein. Analysis was performed with Scion Image software on three different experiments for the panel on the left and two experiments for middle and right panel. E) Densitometric analysis of pERK and pAkt proteins relative to experiments reported in Figure 1J. The intensity of the band relative to pERK or pAkt proteins was normalized to the intensity of the band of the total ERK or total Akt protein. Analysis was performed with Scion Image software on two different experiments. F) Densitometric analysis of caspase 3 protein relative to experiments reported in Figure 1K. The intensity of the band relative to caspase 3 protein was normalized to the intensity of the band of the tubulin protein. Analysis was performed with Scion Image software on two different experiments.

**
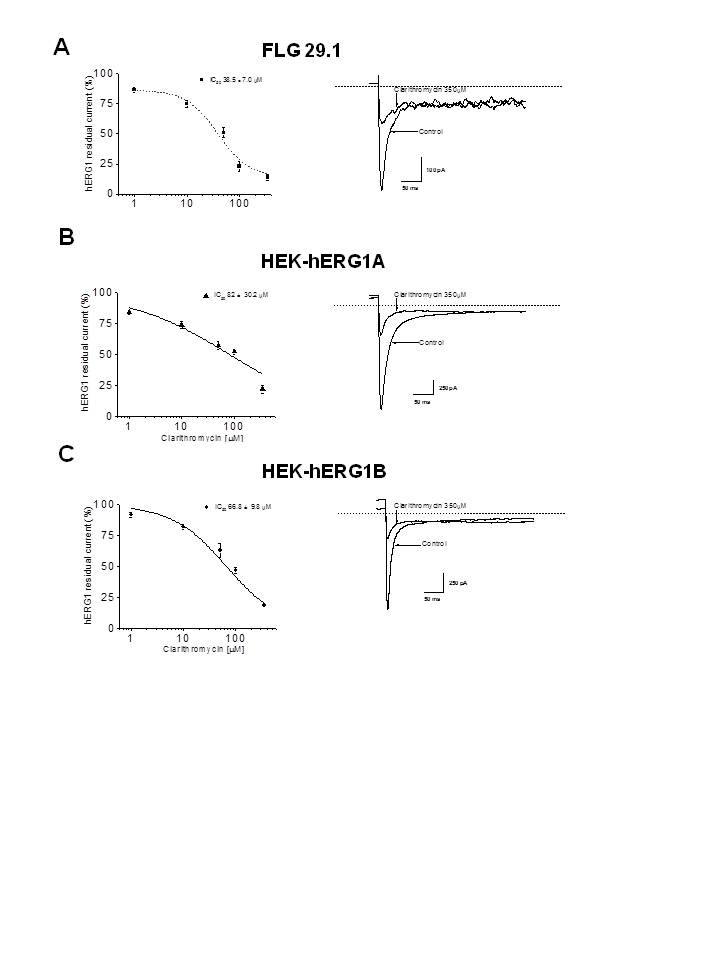
**

**Supplementary Figure S3.** **Effects of Clarithromycin on hERG1 currents.** Concentration-response curves and IC_50_ values after perfusion with different concentrations of Cla, and representative hERG1 currents (in the absence or in the presence of Cla concentrations indicated in the panels) elicited in A) FLG29.1 cells; B) HEK-hERG1A cells; C) HEK-hERG1B cells. For clarity, we reported the drug effect only on the tail currents elicited after conditioning at 0 mV. The inset shows the protocol, detailed in Supplementary informations.

**
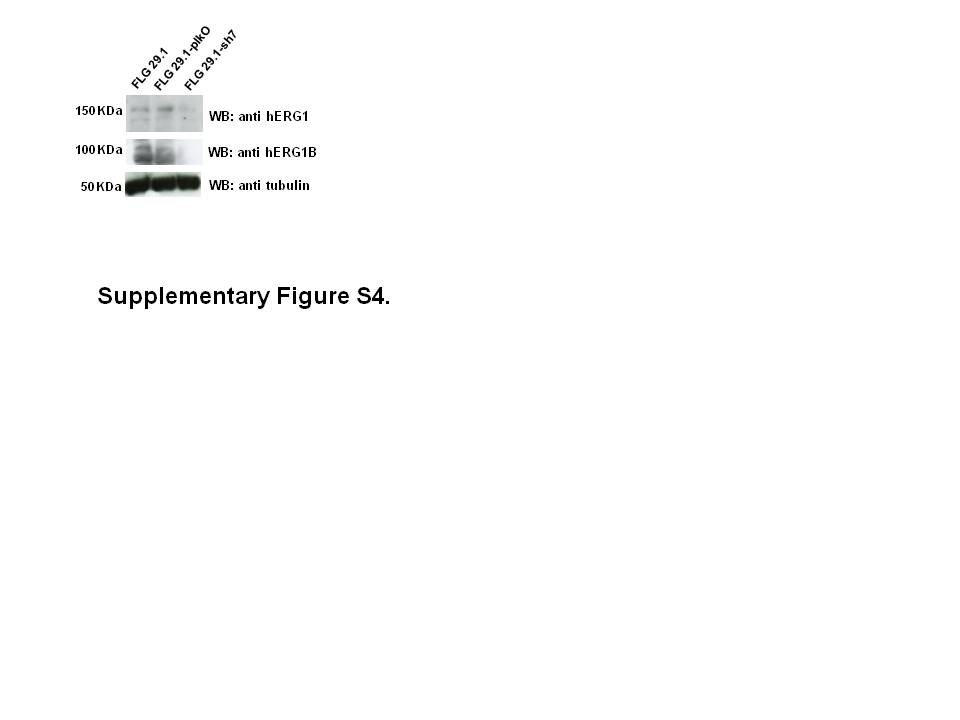
**

**Supplementary Figure S4.** hERG1 proteins expression on FLG 29.1, FLG 29.1-plkO and FLG 29.1-sh7 (hERG1 silenced) cell lines. Cell lysates from cell lines, cultured in the presence of serum, were probed with the anti-pan hERG1 antibody. Reprobing of the membrane with anti tubulin antibody is reported in the bottom panel. Densitometric measurements relative to the blot reported (normalized on the amount of tubulin and expressed as arbitrary units) are the following: hERG1A-FLG 29.1: 1; FLG 29.1-plkO: 1.15; FLG 29.1-sh7:0.55 and hERG1B- FLG 29.1: 1; FLG 29.1-plkO: 0.95; FLG 29.1-sh7:0.24.


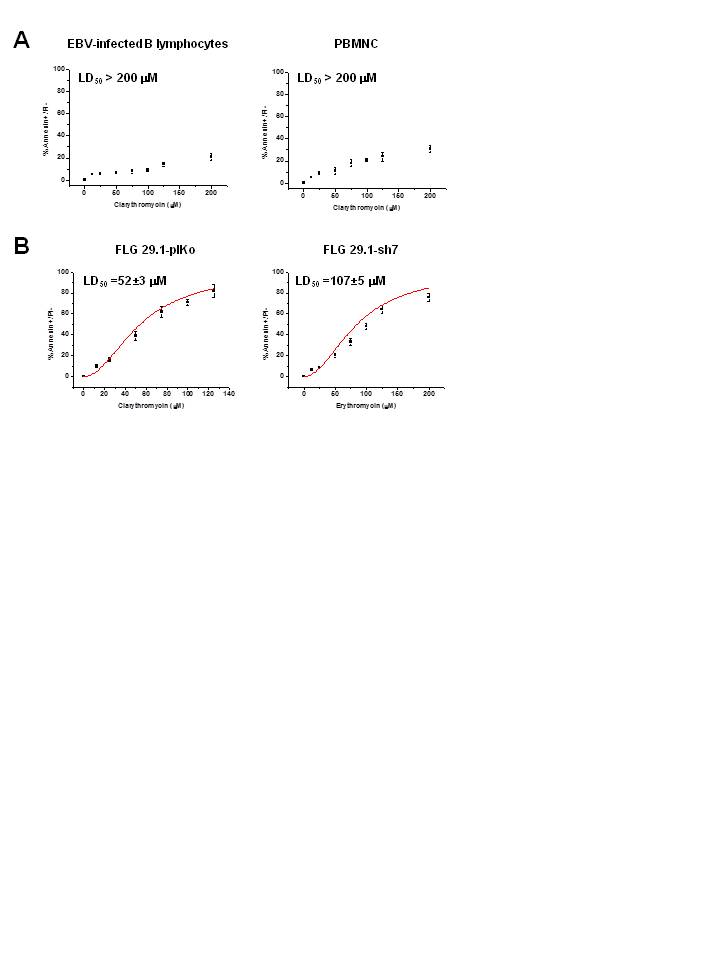


**Supplementary Figure S5. Effects of Clarithromycin on EBV-infected B lymphocytes and on normal PBMNC. Effects of Clarithromycin on EBV-infected B lymphocytes, normal PBMNC and hERG1-silenced leukemia cells.** A) Dose response curves of Annexin V+/PI- cells, and LD_50_ values of Cla in EBV-infected B lymphocytes and PBMNC, after 48 hours of treatment. LD_50_ values were calculated by nonlinear regression analysis using Origin 6 software (Microcal Software). Values are means±SEM of three indipendent experiments each performed in triplicate for EBV-infected B lymphocytes and of two independent experiments for PBMNC (pool of two healthy donors). B) Dose response curves of Annexin V+/PI- cells, and LD_50_ values for Cla in hERG1 silenced leukemic cells FLG 29.1 (FLG 29.1-sh7) and FLG 29.1-plKo cells, after 48 hours of treatment. LD_50_ values were calculated as above. Values are means±SEM of three indipendent experiments.


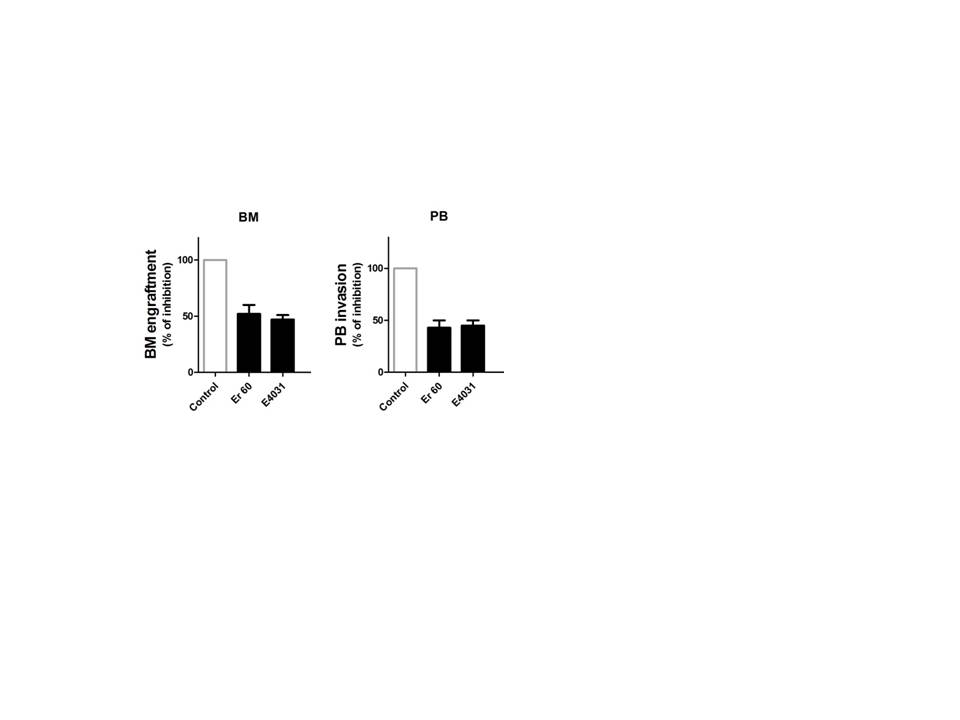


**Supplementary Figure S6. Effects of high dose of MA Er and conventional anti hERG1 blocker in ALL *in vivo.*** NOD SCID mice were inoculated with REH cells on day 0 and after one week treated for two weeks with saline (Con, n=4), E4031 (20 mg/kg, n=4) and Er (Er60, 60mg/Kg, n=4) and sacrificed 3 weeks after cell injection. Leukaemia BM engraftment and PB burden were evaluated by FACS analysis estimating the hCD45+/mCD45+ ratio and were reported as percentage of the control for each treatment group.

**Supplementary Tables**

|  | ***Cell line*** | **Clarithromycin LD_50_** (μM) | **Erythromycin**  **LD_50_** (μM) |
| --- | --- | --- | --- |
| **LYMPHOID** | ***REH*** | 92±3 | 87±4 |
|  | ***697*** | 69±3 | 78±8 |
|  | ***Jurkat*** | 87±6 | 77±5 |
|  |  |  |  |
| **MYELOID** | ***FLG 29.1*** | 56±4 | 77±6 |
|  | ***HL60*** | 37±5 | 82±4 |
|  | ***K562*** | 52±3 | 87±6 |

**Supplementary Table S1. LD_50_ values of Clarithromycin (Cla) and Erythromycin (Er) in a panel of acute leukemia cell lines.** Cells were treated with different concentrations of Cla and Er for 48 hours and analyzed through the Annexin V/PI test. LD_50_ values were evaluated by nonlinear regression analysis using Origin 6 software (Microcal Software). Values are means ± SEM of three indipendent experiments each performed in triplicate.

|  | **Cell line** | **Treatment** | **CI**  **Suspension** | **CI**  **MSC** | ***p*** |
| --- | --- | --- | --- | --- | --- |
| **LYMPHOID** | *697* | Doxo+ Er (LD_50_) | > 1 | 0.865 | *0.039* |
|  |  | Doxo+ Cla (LD_50_) | > 1 | 0.789 | *0.047* |
|  | *REH* | Doxo+ Er (LD_50_) | > 1 | 0.876 | *0.042* |
|  |  | Doxo+ Cla (LD_50_) | > 1 | 0.855 | *0.031* |
|  |  |  |  |  |  |
| **MYELOID** | *HL60* | Cita+Er (LD_50_) | > 1 | 0.656 | *0.022* |
|  |  | Cita+Cla (LD_50_) | > 1 | 0.657 | *0.009* |
|  | *FLG 29.1* | Cita+Er (LD_50_) | > 1 | 0.787 | *0.023* |
|  |  | Cita+Er (LD_30_) | > 1 | 0.992 | *0.033* |
|  |  | Cita+Cla (LD_50_) | > 1 | 0.879 | *0.034* |
|  |  | Cita+Cla (LD_30_) | > 1 | 0.903 | *0.041* |

**Supplementary Table S2. Combination index (CI) values of macrolide antibiotics (MAs) clarithromycin (Cla) and Erythtromycin (at both LD_50_ and LD_30_ concentration, see Table 1S) with doxorubicin (Doxo) and cytarabine (Cyt) (at the LD_50_ dose) in combination in a panel of leukemic cell lines cultured in suspension or onto MSCs.** In the AML cell line FLG 29.1 the synergic interaction between chemotherapy drug and Cla was evident also with dose below LD_50_ (LD30=30 μM). CI values were calculated using CalcuSyn software (Biosoft). The statistical analysis was performed using the Student *t* test comparing CI values in suspension versus CI values on MSC for each drug combination. CI> 1, antagonisms; CI=1, additivity; CI< 1, synergy.
